# Transperineal Laser Ablation (TPLA) Treatment of Focal Low–Intermediate Risk Prostate Cancer

**DOI:** 10.3390/cancers16071404

**Published:** 2024-04-03

**Authors:** Gugliemo Manenti, Tommaso Perretta, Marco Nezzo, Federico Romeo Fraioli, Beatrice Carreri, Paola Elda Gigliotti, Andrea Micillo, Andrea Malizia, Daniele Di Giovanni, Colleen Patricia Ryan, Francesco Giuseppe Garaci

**Affiliations:** 1 Diagnostic Imaging and Interventional Radiology Policlinico Tor Vergata, Department of Biomedicine and Prevention, University of Rome Tor Vergata, 00133 Rome, Italy; 2 Industrial Engineering, University of Rome Tor Vergata Engineering Macro Area, 00133 Rome, Italy; 3 Laboratory of Neuromotor Physiology, IRCCS Fondazione Santa Lucia, 00179 Rome, Italy

**Keywords:** mpMRI, prostate cancer, TPLA, FLA, focal laser ablation, transperineal laser ablation

## Abstract

**Simple Summary:**

Over the past few years, there has been a notable escalation in scholarly attention towards the implementation of focal therapeutic interventions for patients diagnosed with low- and intermediate-risk prostate cancer in order to achieve long-term cancer control and reduce morbidity associated with surgery and radiation therapy. This interventional pilot study (ClinicalTrials.gov ID NCT04045756) aimed to evaluate the short/medium-term (3 years) efficacy of trans perineal laser ablation (TPLA) in treating the index lesion of low–intermediate-risk prostate cancer, along with assessing the safety of the procedure and Quality of Life (QoL) of patients. Our results suggested that TPLA is a safe, feasible and effective treatment of low–intermediate-risk prostate cancer, with a high rate of tumor eradication and preservation of Quality of Life.

**Abstract:**

**Background:** This interventional pilot study aimed to evaluate the short-term (3 years) efficacy of focal laser ablation (FLA) in treating the index lesion of low–intermediate-risk prostate cancer, along with assessing the safety of the procedure (ClinicalTrials.gov ID NCT04045756). **Methods:** Forty patients aged between 46 and 86 with histologically proven organ-confined prostate cancer and low-to-intermediate progression risk were included. FLA was performed under percutaneous fusion magnetic resonance/ultrasound guidance in a Day Hospital setting under local anesthesia. Patients underwent regular clinical and functional assessments through the international index of erectile function (IIEF-5) and the International Prostatism Symptom Score (IPSS), PSA measurements, post-procedure MRI scans, and biopsies at 36 months or if positive findings were detected earlier. Statistical analyses were conducted to assess trends in PSA levels and cavity dimensions over time. **Results:** Forty patients were initially included, with fifteen lost to follow-up. At 36 months, a mean PSA reduction of 60% was observed, and 80% of MRI scans showed no signs of in-field clinically significant residual/recurrent cancer. Biopsies at 36 months revealed no malignant findings in 20 patients. No deterioration in sexual function or urinary symptoms was recorded. **Conclusions:** FLA appears to be safe, feasible, and effective in the index lesion treatment of low–intermediate-risk prostate cancer, with a high rate of tumor eradication and preservation of quality of life.

## 1. Introduction

Prostate cancer (PCa) is the second most common neoplasm diagnosed in men worldwide [1].

Approximately half of the patients affected by PCa are found to have a low-risk progression PCa with a maximum Gleason score of 6 (Grade Group 1) and with a mortality rate of 0.2% after 20 years [2].

Furthermore, even in patients affected by Grade 2 prostate cancer (Gleason score 3 + 4), survival rates are not significantly different than in patients with Grade Group 1, despite the elevated risk of disease progression [3].

Current standard therapy for PCa with radical intent usually consists of radical prostatectomy or radiotherapy, plus androgen deprivation therapy (ADT). Unfortunately, treatment-related morbidity is high. The ProtecT trial showed urinary incontinence rates of 3% and 20% and erectile dysfunction rates of 66% and 79% for radiotherapy and radical prostatectomy, respectively [4].

Consequently, over the past few years, there has been a notable escalation in scholarly attention towards the implementation of focal therapeutic interventions for patients diagnosed with low- and intermediate-risk prostate cancer (PCa). This heightened focus has fostered the exploration and development of several focal treatment modalities, including high-intensity focal ultrasound (HIFU), irreversible electroporation, focal cryotherapy, and photodynamic therapy [5].

According to the European Association of Urology (EAU), low-risk and intermediate-risk patients may undergo local procedures, with the aim of achieving long-term cancer control and reducing morbidity associated with surgery and radiation therapy [5].

Notwithstanding the considerable diversity in treatment techniques, it is noteworthy that each of these modalities is associated with a notable incidence of persistent cancer within the prostate upon re-biopsy, ranging from 24% to 49%. Importantly, these treatments are characterized by minimal occurrences of high-grade complications. Instances of incontinence are rare, and any reductions in erectile function are typically mild and transient. The most common adverse events are urinary tract infections, hematuria, and urinary retention [6].

Focal laser ablation (FLA) is a safe and effective treatment of focal cancer lesions, not only prostatic; this is due to the very thin caliber of the applicators and the high precision of the delivered energy [7,8,9]. This procedure involves a minimally invasive percutaneous thermoablation technique, which utilizes a 1064 nm laser light delivered into the target tissue through optical fibers for a brief duration, resulting in the heating of the affected tissue to the point of irreversible, in situ damage, thus inducing coagulative necrosis.

Recently, several pilot studies showed that transperineal laser ablation (TPLA) is feasible for the treatment of PCa under local anesthesia [10,11,12].

In our study, we enrolled patients with prostate cancer (PCa) who exhibited intermediate-risk progression (defined as PSA levels below 20 ng/mL, Gleason Scores of 3 + 4 or 4 + 3, and clinical staging between cT2a and cT2b). Additionally, we included patients with low-risk progression (characterized by PSA levels below 10 ng/mL, Gleason score 3 + 3, and clinical staging ranging from cT1 to cT2a). These patients expressed a preference to forego or they declined participation in active surveillance protocols, as well as validated treatments for organ-confined cancer, such as radical surgery and radiotherapy.

Our primary objectives encompassed a thorough assessment of the short-term (over a 3-year period) efficacy of magnetic resonance imaging (MRI)/ultrasound (US) fusion-guided focal laser ablation (FLA) for the treatment of low-to-intermediate-risk prostate cancer, as well as an evaluation of treatment-related complications. The effectiveness of this intervention was quantified by gauging cancer control, defined as the proportion of patients achieving disease-free survival, as determined by target biopsy outcomes.

Concurrently, our secondary aim involved an appraisal of the utility of multiparametric magnetic resonance imaging (mpMRI) as a diagnostic tool during post-treatment follow-up to examine the morphostructural alterations in the prostate gland resulting from FLA. Additionally, we assessed its reproducibility in determining the response to FLA therapy, correlating these findings with biopsy results, with the ultimate objective of establishing its predictive diagnostic value in identifying residual or recurring tumors.

## 2. Materials and Methods

Starting in 2018, we enrolled a cohort of forty male patients, aged between 46 and 86, all of whom were diagnosed with histologically confirmed, organ-confined prostate cancer displaying low-to-intermediate progression risk (as defined by Gleason scores ≤ 4 + 3, PSA levels below 20 ng/mL, and clinical staging below T2b).

Ethical approval for all research procedures was obtained in accordance with the ethical standards of the institutional and/or national research committee, and in accordance with the guidelines stipulated in the 1964 Helsinki Declaration and its later amendments (ClinicalTrials.gov ID NCT04045756).

The study unfolded across seven distinct phases. At Time 0, our focus was on patient selection, involving the recruitment process and a rigorous assessment of eligibility criteria. This step was undertaken by the Multidisciplinary Neoplasms Group specializing in uro-genital cases within our hospital. Patients were evaluated and recruited based on the inclusion and exclusion criteria summarized in Table 1.

**Figure 1:**
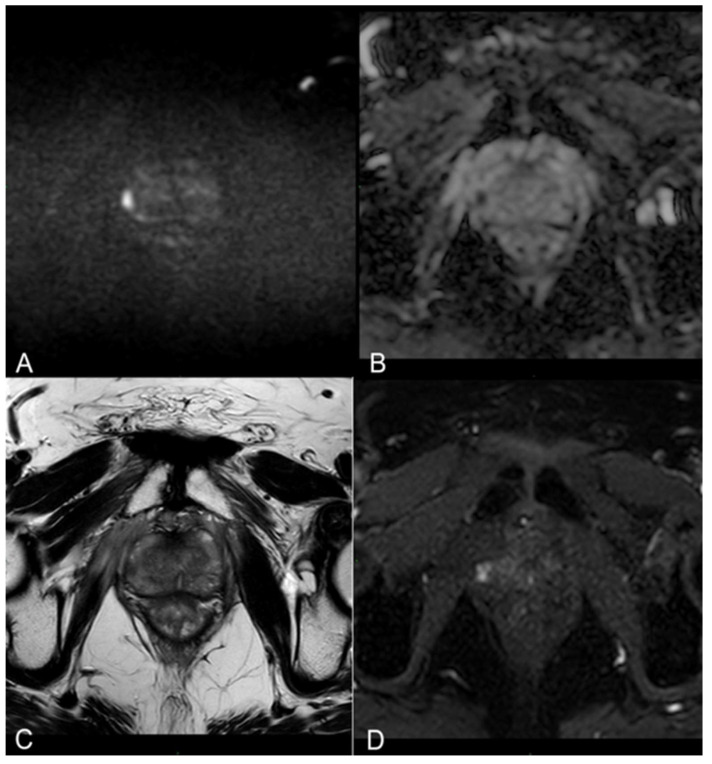
(**A**,**B**) Diffusion-weighted imaging (DWI) b-value 2500 s/mm^2^ and ADC map on axial plane showing focal marked hyperintensity on high b-value DWI sequences corresponding to a hypointensity on the ADC map (PI-RADS 4). (**C**) T2-weighted TSE sequence on axial plane showing a hypointense lesion in the peripheral zone of the right middle portion of the gland. A curvilinear contact surface with the prostate capsule is demonstrated without any sign of capsule invasion. (**D**) Dynamic Contrast-Enhanced Imaging (DCE) with T1-weighted Dixon sequence with fat suppression on axial plane: early and significant focal enhancement in the peripheral zone of the right mid-portion of the gland.

The pre-procedural evaluation consisted of an electrocardiogram, complete blood count, urine examination and urine culture, and evaluation of post-voiding residue and uroflowmetry. Patients were also submitted to IPSS-QoL and IIEF-5 questionnaires at baseline and during follow-up to investigate any procedure-related erectile dysfunction or urinary symptoms. Given that the procedure was guided by MRI/US fusion images, a 3T multiparametric prostate resonance (mpMRI) scan was performed for each patient at Time 0, adhering to the protocol delineated in Supplementary Table S1 (refer also to Figure 1).

Time 1: Interview and signing of informed consent.

Time 2: FLA treatment.

The procedure was performed by an expert radiologist on prostate MRI/US procedures, in a Day-Hospital setting, in a radiological interventional room. The patients were placed in the lithotomy position, and Ciprofloxacin 500 mg was administered as antibiotic prophylaxis. The Echolaser system (Elesta s.r.l.—Calenzano (FI)) as laser light source operating at 1064 nm and MRI/US fusion imaging to identify the focal lesion to treat were used. Local superficial anesthesia of the perineal region, followed by a transrectal prostatic block with a lidocaine solution 2% (20 mL), was performed under US guidance. The laser light was conveyed from the source to the target through flexible, small-caliber (300 micron), flat-tipped quartz optical fibers introduced transperineally by thin needles (21 Gauge) inserted previously under real-time US guidance. Up to three needles were used according to the lesion size because the multi-fiber approach can extend the coagulation area. The laser fiber protrudes 10 mm from the needle tip. Each lighting lasted about 6 min, and a maximum energy of 3600 J per fiber was delivered (1800 J per fiber with the pull-back technique, which consists of pulling back the introducer by 1 cm and performing a second illumination to increase the ablation area with duration and power equal to the previous one) with a power of 3–5 Watts. The laser therapy was performed entirely under US guidance for the real-time monitoring of the correct positioning of applicators and the extension of the area of damage.

An mpMRI was performed immediately after the treatment to evaluate the extent of the ablation zone according to the protocol in Supplementary Table S1. At the end of the procedure, all patients were observed for a minimum period of 2 h. The resumption of spontaneous diuresis was checked out. No cases of acute urinary retention or clinically significant hematuria that required a bladder catheter insertion occurred.

An oral cortisone drug as anti-edema therapy was prescribed.

Patients were discharged with antibiotic therapy, given pain-relief drugs if needed, and gastroprotective for 7 days.

Time 3–4–5–6–7: follow-up at 1 (T3), 6 (T4), 12 (T5), 24 (T6), and 36 (T7) months with clinical evaluation performed by PSA measurement and mpMRI. At 36 months, the IPSS and IIEF-5 questionnaires were repeated, and a systematic and target MRI/US fusion-guided prostate biopsy was performed.

Treatment success was operationally defined as the absence of any anomalies in MRI examinations conducted during the follow-up period, in conjunction with a negative target biopsy at the conclusion of the 36-month period and evaluation of serum PSA. The presence of residual lesions within the treatment field was regarded as an indicator of treatment failure. Notably, a positive random biopsy indicating the existence of out-of-field lesions was not construed as a treatment failure.

### Statistical Analysis

A statistical analysis was realized on the cohort of 20 patients treated with TPLA. It was performed using Visual Basic (VB) as code, and Excel has a platform to create graphs and tables to investigate changes over time in PSA and necrotic cavity volume during the 36-month follow-up (as seen, respectively, in Supplementary Tables S2 and S3) after the procedure. Different fitting algorithms to obtain equations predicting the trend of the variables over the 36 months were tested. The tests were completed for the first 3 patients with some trendline options: linear, logarithmic, polynomial, power, moving average, and exponential. The exponential trendline showed a better fit with the data, and it was applied to all the patients, both for PSA and cc cavity. In Supplementary Tables S4 and S5, you can find the trend equations and the related R^2^. Additional statistical tests (e.g., Kolmogorov–Smirnov test and *t*-test) were performed using IBM SPSS statistics, version 27 (IBM SPSS, IBM Corp., Armonk, NY, USA), and a *p*-value < 0.05 indicated a statistically significant difference and was calculated accordingly to the literature.

We evaluated clinical data and QoL variations of patients through the IPSS score (Supplementary Table S6) and the IIEF-5 score (Supplementary Table S7), measured at times 0 and 36 months, respectively. Functional outcome was evaluated by comparing IPSS and IIEF-5 mean values at baseline with those at 36 months after using Student’s *t*-test.

## 3. Results

All procedures were executed with technical proficiency, ensuring successful completion in all instances. Following their treatment, all patients were discharged on the same day.

The clinical follow-up entailed the monitoring of potential complications associated with focal laser ablation (FLA), including perineal pain, perineal hematoma, hematuria, acute urinary retention, hematospermia, erectile dysfunction, and urinary incontinence. It is noteworthy that none of these complications were observed during the follow-up and clinical observation, signifying a lack of any such occurrences and, thus, aligning with the Clavien–Dindo grade I classification.

### 3.1. Oncological Outcomes

We assessed the temporal changes in PSA levels among our cohort.

**Figure 2:**
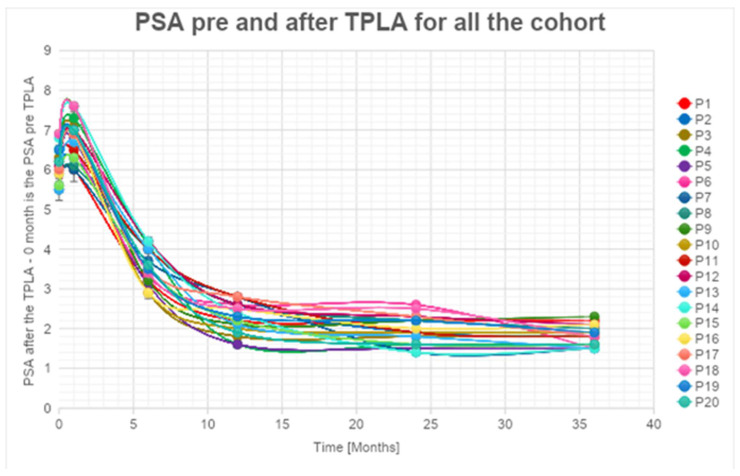
PSA levels are reduced during follow-up.

Notably, at the 1-month follow-up, every patient exhibited an increase in PSA levels compared to the baseline, attributed to the release of enzymes into the bloodstream following the necrotic process. However, subsequent to this initial increase, we observed a consistent and statistically relevant decline in PSA levels, with a mean reduction exceeding 60% at the 36-month follow-up for all patients (Supplementary Table S2, Figure 2).

A significant decrease in PSA values higher than 55% at 1 year after the treatment (with a peak of 75%) for PSA (Supplementary Table S2, Figure 2) was observed. These percentages increased and are always higher than 60% (with a peak of 80%) 2 years after the treatment. The percentage of decrease in PSA values is always lower than the 5% between the 2nd and 3rd years. We can affirm that the decrease is significant in the first 24 months. An estimated error of 5% during the data acquisition phase is considered.

The exponential trendlines show an estimated decrease higher than 87% for all the patients observed at 48 months from the treatment.

It is noteworthy that five out of 25 patients did not exhibit a substantial decrease in PSA levels during follow-up. Subsequent MRI and target biopsy identified the presence of a residual lesion in close proximity to the treatment area in these cases.

An imaging follow-up through 3T mpMRI was performed.

**Figure 3:**
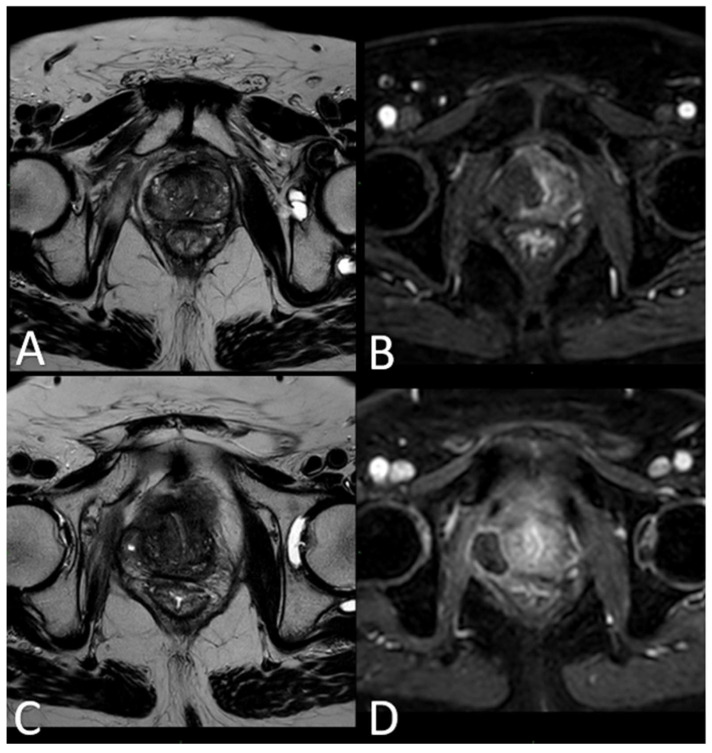
(**A**) T2-w TSE sequence on axial plane 1 h after the treatment: an elliptical ablation cavity superimposing the lesion, filled with blood derivates and fluid as a result of tissue ablation. (**B**) DCE T1-w Dixon sequence 1 h after the treatment: unenhanced cavity in the right peripheral zone. (**C**) T2-w sequence on axial plane at 1-month follow-up: much wider ablative lesion with core charring, a hyperintense thick rim of necrotic tissue with an outer hypointense thin rim of hemorrhage. Fiber tracks are visible as hyperintense spots. Bulging of the capsule and liponecrosis on the right recto-prostatic triangle. (**D**) DCE T1-w Dixon sequence on axial plane at 1-month follow-up: unenhanced ablation cavity with a thick margin.

Immediate post-treatment mpMRI examinations consistently revealed the presence of a devascularized ablation cavity characterized by coagulative necrosis in all cases, and notably, this cavity exhibited a volume approximately three times larger than the original cancer lesion (as illustrated in Figure 3).

### 3.2. mpMRI-Derived Results

**Figure 4:**
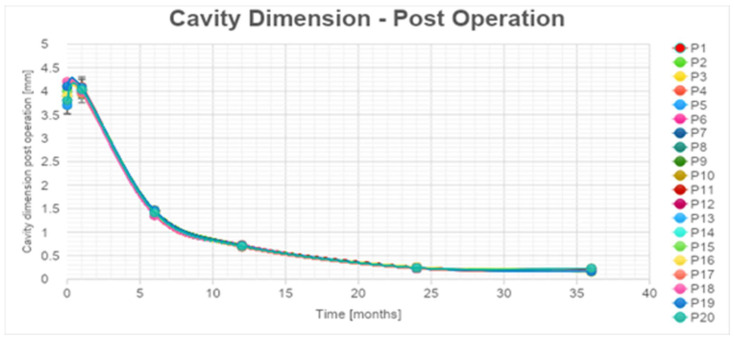
Necrotic cavity volume reduction during follow-up.

Subsequent mpMRI assessments conducted during the follow-up period portrayed the temporal evolution of this cavity, which began to exhibit a noticeable reduction in size after the 6-month follow-up, with an average volume decrease exceeding 80% by the 36-month mark (Supplementary Table S3, Figure 4).

According to the statistical analysis, there was a significant decrease also in cavity volume values that were, respectively, always between 80 and 83% for all the patients at 1 year after the treatment (Supplementary Table S3, Figure 4). The percentages of cc cavity decreasing are always between 93 and 95% 2 years after the treatment. The percentage of decrease in cc cavity values is always lower than the 3% between the 2 and the 3 years. The decrease is considered significant in the first 24 months. An error of 5% during the data acquisition phase was estimated.

For the cavity volume, the exponential trendlines show an estimated decrease higher than 98% for all the patients 48 months after the treatment.

**Figure 5:**
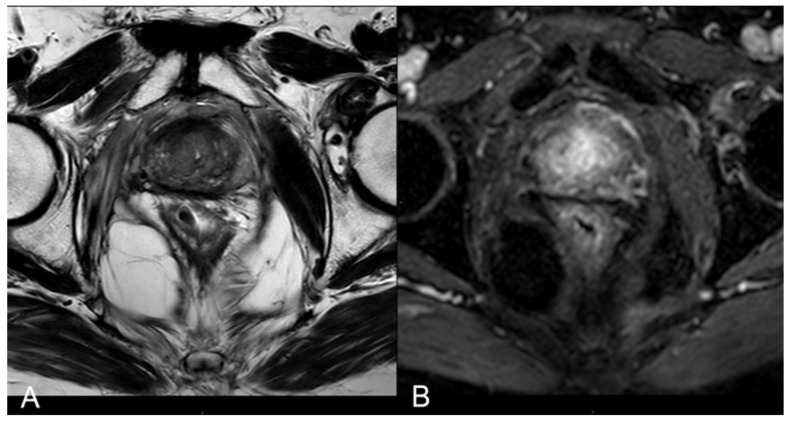
(**A**) T2-w axial sequence: small cavity filled with fluid. Some fibrotic tissue on the posterior edge of the right gland, with an external capsule retraction. Band-like right-side recto-prostatic angle hypointensity on T2-w imaging is the effect of mesorectum liponecrosis. (**B**) DCE T1-w Dixon sequence with fat suppression on axial plane: millimetric unenhanced cavity resembling a small cavity filled with proteinaceous fluid. Necrotic tissue is reabsorbed. Capsule retraction is visible.

In 25% of the cases, the necrotic coagulation cavity was entirely reabsorbed and replaced by a fibrotic scar, as exemplified in Figure 5. Among the 25 patients, 20 did not exhibit any reliable focal contrast enhancement indicative of residual or recurrent neoplastic lesions within the treated area. This absence of neoplasia was subsequently confirmed by MRI/US fusion-guided target biopsies conducted at the 36-month follow-up.

### 3.3. Functional Outcomes

Importantly, although already known in the literature [8], the IIEF-5 and IPSS scores demonstrated non-statistically significant variations (*p*-value > 0.05) between the pre-procedural values and those recorded at the 36-month mark (as described below in the paragraph on the statistical analysis results; Supplementary Tables S6 and S7).

The mean IPSS scores PRE and 36 months after the procedure decreased from 7.35 to 6.70. The difference between mean IPSS scores PRE and POST (after 36 months) analyzed with a *t*-test for paired samples resulted in a t-statistic of approximately 1.82, with a *p*-value of 0.085 (Table 2). This *p*-value is above the conventional significance level of 0.05, suggesting that there is no statistically significant difference between IPSS scores before and 36 months after. Erectile function, which was measured by the mean PRE IIEF-5 scores and 36 months after the procedure, increased from 14.20 to 15.50. The difference between mean IIEF-5 scores PRE and POST (after 36 months) analyzed with a *t*-test for paired samples yielded a t-value of approximately −1.70, with a *p*-value of 0.105 (Table 2). This *p*-value is above the conventional significance level of 0.05, suggesting that there is no statistically significant difference between IIEF-5 scores before and 36 months after.

However, for the remaining 5 out of 25 patients, the mpMRI at the 12-month follow-up identified a focal lesion adjacent to the ablation cavity in all cases (as depicted in Figure 4), promptly confirmed by target biopsy. In all of these instances, the biopsy results indicated a Gleason Score of 3 + 4 or 4 + 3 neoplasm (Table 3). Given the proximity of these lesions to the necrotic cavity, they were all interpreted as being indicative of residual cancer.

## 4. Discussion

Currently, a myriad of focal treatment modalities is available for the ablation of prostate cancer. These encompass cryotherapy, high-intensity focused ultrasound (HIFU), irreversible electroporation, photodynamic therapy, and focal brachytherapy [13].

Compared to focal laser ablation (FLA), cryotherapy necessitates the use of larger needle calibers and deploys multiple probes situated within the gland, strategically positioned between the prostate and the rectum for temperature monitoring. Additionally, a transurethral warming device is employed to safeguard against urethral damage. However, a drawback of cryotherapy lies in the challenge of precisely controlling the size of the ice ball, often leading to a wider treatment area than required, which can inadvertently result in the ablation of the ipsilateral neurovascular bundle [14,15].

HIFU is a relatively new treatment option for prostate cancer, and limited data are available regarding its safety. It requires general anesthesia and a transrectal approach, being then able to treat only posterior lesions [6,16,17,18].

Photodynamic therapy (PDT) shares some similarities with FLA, as it also employs transperineally inserted optical fibers directed at the prostate target. However, it is noteworthy that Azzouzi et al. reported a notable rate of 38% for erectile dysfunction and urinary complications associated with PDT [13].

A study on the irreversible electroporation investigated 63 patients and reported a 16% of in-field recurrence with a mild decline in the sexual quality-of-life score [13]. Within focal brachytherapy, seeds are transperineally inserted into the prostate. King et al. conducted an evaluation of 354 patients who underwent brachytherapy focused on the peripheral zone, revealing long-term oncologic results that are concerning. Specifically, they reported a 10-year biochemical progression-free survival rate of only 28% for patients with intermediate-risk prostate cancer [13].

Focal laser ablation (FLA) represents a minimally invasive percutaneous procedure that leverages laser light transmitted through optical fibers to selectively elevate the temperature within tissue. This thermal intervention results in protein denaturation and the induction of irreversible coagulative necrosis. Notably, FLA is versatile in its application, as it is capable of ablating cancer lesions in any region of the prostate. Furthermore, it offers the advantage of MRI compatibility, enabling real-time, in-bore MRI guidance during the procedure [16,19].

Moreover, this approach can be performed on an outpatient basis and under local anesthesia. The limited side-effect profile due to the excellent precision of the ablation obtainable with laser energy delivery is a great advantage of FLA [20].

The effectiveness of focal laser ablation (FLA) has been previously documented in studies focusing on the treatment of benign prostatic hyperplasia (BPH) and benign thyroid and hepatic nodules [7,9,21,22,23].

There are few studies about FLA, and most of them have a small sample size and short follow-up.

In a 2016 published phase II trial, Eggener et al. treated 27 men with a Gleason 7 or less prostate cancer. At 3 and 12 months, they found, respectively, a 4% and 11% percentage of disease recurrence within the ablation zone (in-field recurrence). However, 37% of patients were found to have some residual cancer within the prostate gland on a systematic 12-cores re-biopsy (out-of-field recurrence) performed at 12 months [6,10,19].

Lepor et al. published their results of 25 consecutive patients with low–intermediate-risk PCa treated with MRI-guided FLA [24,25]. The post-ablation biopsy at 3 months showed no evidence of cancer in 96% of the patients, without a compromise in the functional outcomes.

In 2018, interim findings from the most extensive study evaluating focal laser ablation (FLA) were disclosed (Feller et al.) [20]. This study encompassed the treatment of 98 patients and addressed 138 tumor foci, all performed under real-time MRI guidance. Their report highlighted a 23% rate of in-field cancer recurrence, a notable outcome; and, reassuringly, there were no reports of serious adverse events.

Forty cases of low-to-intermediate progression risk prostate cancer were selected and treated with focal laser ablation in our hospital. Multiparametric magnetic resonance imaging (mpMRI) was used for the initial assessment of the lesions, pre-procedural planning, and post-treatment imaging follow-up. Ultrasound/magnetic resonance (US/MRI) fusion-guided biopsies were performed at 36 months. In five cases, the biopsy was performed between 12 months and 24 months because the mpMRI showed an in-field recurrence.

Throughout the follow-up period, no patients exhibited statistically significant alterations in urinary symptoms or erectile dysfunction.

In almost all patients, we witnessed stability or even a slight improvement in urinary symptoms and sexual function over time; in cases of worsening, however, patients were seen to fall into the same severity class. This finding, although already described in the short term in the literature [8], is particularly important because it is the first time it has been evaluated in the medium-to-long term (36 months after the procedure).

This favorable outcome can be attributed to several reasons: first of all, the specific location of the lesions, which were consistently situated within the peripheral zone of the prostate; and the operator’s expertise. In addition, a psychological factor cannot be excluded. Consequently, the procedure effectively preserved the integrity of the urethra and the bladder neck in all cases.

One month after the procedure, there was a transient increase in PSA levels. This is a common occurrence after localized prostatic procedures attributed to the sudden release of PSA into the bloodstream by necrotic cells, and it is considered a favorable prognostic factor [26]. Beginning in the third month of the follow-up period, a notable decline in PSA levels became evident. By the time we reached the 36-month mark, we had observed a consistent and statistically significant reduction of over 60% in PSA levels.

We attained an impressive 80% success rate with the procedure, encountering only five cases with residual lesions. Remarkably, these patients opted for radical prostatectomy as a salvage treatment option, even though a second FLA could have been a viable alternative. Another notable advantage of FLA is its flexibility, allowing patients to explore more invasive and well-established treatments in the event of treatment failure. Importantly, throughout the entire 36-month follow-up period, we did not record any instances of complications or side effects linked to the procedure. The transperineal approach, which safeguards the urethra and rectum, played a pivotal role in minimizing potential complications.

While other studies on the subject of FLA have been conducted, to the best of our knowledge, our study represents a pioneering effort in investigating focal laser ablation (FLA) for the treatment of low-to-intermediate-risk prostate cancer. A distinctive feature of our study is the consistent utilization of multiparametric magnetic resonance imaging (mpMRI) as an imaging tool throughout the follow-up period, which distinguishes it from previous research in this field. The use of mpMRI as the primary follow-up imaging method resulted in high accuracy for the validation of the procedure [27,28,29].

We conducted a qualitative and quantitative evaluation of the main imaging findings at each follow-up step.

Immediately following the procedure, we observed elliptical hypointense ablation cavities that were roughly three times the size of the original lesions. Some of these cavities contained fluid and/or complex fluid (comprising blood and proteinaceous material), with hyperintense fiber tracks discernible within the treated area (as depicted in Figure 3A,B).

**Figure 6:**
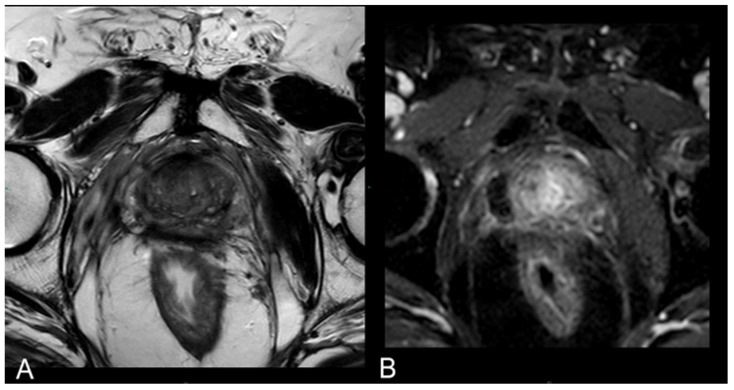
(**A**) T2-w sequence on axial plane: coagulation necrosis area (hypointense in T2W imaging) is smaller because of necrotic tissue reabsorption. (**B**) DCE T1-w Dixon sequence: The unenhanced devascularized area (hypointense in T1W + C imaging) is smaller in size compared with the 1st-month MR follow-up.

At 1 month–6 months–12 months: The main findings included the laser fiber tracks surrounded by a large elliptical-shaped necrotic tissue cavitation, which appeared hypointense on T2-weighted images (Figure 3C,D and Figure 6).

At 24 months–36 months: presence of a T2W hypointense scar tissue that almost completely replaced the original cavity (Figure 5).

These imaging characteristics are similar to those documented in the TPLA of BPH [7].

We observed a progressive decrease in ablation cavity volume, and by 36 months, it was less than 70% in all patients. It would be helpful to continue the follow-up of patients to determine if the decreasing trend in PSA levels and ablation cavity volume remain stable.

In our experience, we reported 10 cases, accounting for 40% of patients, where new prostate cancer foci emerged outside the ablation zone. Importantly, these newly identified lesions consistently exhibited a Gleason Score of 3 + 3. Their presence was ascertained through the performance of random biopsies at the 36-month follow-up. Notably, the multiparametric magnetic resonance imaging (mpMRI) yielded negative results for all of these cases, indicating the absence of clinically significant lesions.

To establish more robust statistical analyses, it would be advantageous to augment the patient cohort and potentially broaden the selection criteria. Similar to other innovative interventional techniques, focal laser ablation (FLA) mandates the expertise of trained operators. In our study, the operators possessed approximately 10 years of experience, which significantly contributed to reduced procedural durations and shorter hospital stays for the patients.

A further limitation of our study lies in the absence of an initial sample size calculation. Instead, we exclusively included patients who met our specified criteria, resulting in a relatively limited sample size. Consequently, our study can be regarded as an initial experience. Therefore, it is imperative to initiate multicenter investigations involving a more extensive patient pool and standardized selection criteria to substantiate FLA’s efficacy as a standard treatment approach for low-to-intermediate-risk prostate cancer.

## 5. Conclusions

Using surrogate parameters such as serum PSA monitoring and MRI follow-up to detect recurrence, we achieved a disease-free rate of 80% over a 3-year period and the absence of any complications among our patients. The study demonstrates the effectiveness and safety of focal laser ablation (FLA) for the treatment of low-to-intermediate-risk prostate cancer in the short-term period. Furthermore, our findings also attest to the favorable short- and mid-term quality of life and functional outcomes associated with FLA.

The full alignment between MRI and histological findings, along with the high accuracy of MRI in assessing morphological changes in the prostate resulting from the procedure, underscores the optimal positive/negative predictive diagnostic value of this imaging modality in the context of residual/relapsing tumors and loco-regional complications.

However, it is imperative to acknowledge that further research and more extensive studies are requisite to validate these outcomes and establish the long-term efficacy and safety of transperineal prostate laser ablation (TPLA) as a viable treatment option for low-to-intermediate-risk prostate cancer.

## Figures and Tables

**Table 1:** Inclusion and exclusion criteria.

Inclusion Criteria	Exclusion Criteria
Male	Gleason score > 4 + 3
Over 45 years old	Clinical stage > T2b
Prostate cancer histologically proven by US/MRI fusion biopsy with target and systematic samples (>12)	PSA > 20 ng/mL
Low risk of progression cancer (Gleason Score 3 + 3, PSA < 10 ng/mL, cT1–cT2a) in patients who refuse or wish to leave active surveillance protocols and who refuse other validated treatments	Presence of metastases detected by imaging
Intermediate risk of progression cancer (Gleason Score 3 + 4 or 4 + 3, PSA < 20 ng/mL, cT2b)	Coagulation disorders
	Inadequate compliance
	Contraindications to MRI
	Paramagnetic contrast agent allergy
	Acute and/or chronic renal failure

**Table 2:** The *t*-test comparing mean IPSS and IIEF-5 before and 36 months after the procedure. Mean values are compared with the previous measurements.

Score	0 Month Mean	36 Months Mean	T	*p*-Value
IPSS	7.35	6.7	1.818	0.085
IIEF-5	14.2	15.05	−1.704	0.105

**Table 3:** Cohort patients with in-field recurrence data.

Patient	Gleason score at baseline	Gleason score of recurrence	Psa at baseline	Psa at the diagnosis of recurrence
Patient 1	4 + 3	3 + 4	9.6 ng/mL	7.5 ng/mL
Patient 2	3 + 4	3 + 4	11.3 ng/mL	9.5 mL
Patient 3	4 + 3	4 + 3	6.75 ng/mL	7.1 ng/mL
Patient 4	3 + 3	3 + 4	4.5 ng/mL	5.7 ng/mL
Patient 5	3 + 3	4 + 3	2.61 ng/mL	3.49 ng/mL

## Data Availability

The raw data supporting the conclusions of this article will be made available by the authors upon request.

## Supplementary Materials

The following supporting information can be downloaded at https://www.mdpi.com/article/10.3390/cancers16071404/s1, Table S1: mpMRI protocol; Table S2: PSA trend during follow-up; Table S3: Necrotic cavity volume trend during follow-up. Table S4: PSA trendline equations and R^2^. Table S5: Cavity cc volume trendline equations and R^2^. Table S6: IPSS score. Table S7: IEFF-5 score.
