# Supplementary material for: Transperineal Laser Ablation (TPLA) Treatment of Focal Low–Intermediate Risk Prostate Cancer
Source: Cancers (Basel). 2024 Apr 3;16(7):1404. doi: 10.3390/cancers16071404 (PMC11011049; doi:10.3390/cancers16071404)
Supplement: Supplementary file 1 [file cancers-16-01404-s001.zip › Supplementary materials.pdf]

Supplementary materials

Table S1. mpMRI protocol

SEQUENCE	PLANE	TIME	TR	TE	SLICES	THICKNESS	PIXEL
T2 TSE	SAG	2:45 min	3293 ms	110 ms	25	3 mm	0.7x0.9 mm
T2 TSE	AX	5:42 min	3000 ms	110 ms	24	3 mm	0.6x0.9 mm
DWI	AX	6:24 min	3459 ms	88 ms	24	3 mm	2.4x2.8 mm
T2 TSE	COR	5:06 min	3000 ms	110 ms	25	3 mm	0.5x0.8 mm
T1 DIXON DYNAMIC (30 phases)	AX	3:35 min	3.7 ms	1.37 ms	25	3 mm	1.5x1.5 mm
T1 TSE	AX	2:21 min	561 ms	8 ms	31	5 mm	1.0x1.0 mm

Table S2. PSA trend during follow-up.

**Patients**	**0 month** PSA	**1 month** PSA	**6 months** PSA	**12 months** PSA	**24 months** PSA	**36 months** PSA
P1	5,5	6	3,2	2,2	2,2	2,2
P2	5,9	6,3	4	2,6	1,9	1,8
P3	6,3	7,1	2,9	1,8	1,8	1,8
P4	6,5	7,3	4,1	1,6	1,6	1,6
P5	6	6,8	3,1	1,6	1,5	1,5
P6	6,1	6,5	3,3	2,6	2,6	1,5
P7	5,6	6	3,7	2,8	1,4	1,5
P8	5,5	6,1	4	2,6	2,2	2
P9	6,9	7,6	3,2	2,1	2,2	2,3
P10	6,2	6,9	2,9	2	1,9	1,9
P11	6,2	6,5	4	2,8	1,9	1,8
P12	6,1	7	4,2	2,6	2,3	2,1
P13	5,5	6,7	4	2,1	1,8	1,5

P14	6,8	7,6	4,2	2,4	1,4	1,5
P15	5,6	6,3	3,5	2,2	1,6	1,6
P16	5,9	6,9	2,9	2,5	2	2,1
P17	6	6,9	3,5	2,8	2,3	1,8
P18	6,9	7,6	3,4	2,5	2,5	1,8
P19	6,5	7	3,5	2,3	2,2	1,9
P20	6,2	7	3,6	1,9	1,6	1,6

Table S3. Necrotic cavity volume trend during follow-up.

**Patients**	**0 month** cc cavity [cm]	**1 month** cc cavity [cm]	**6 months** cc cavity [cm]	**12 months** cc cavity [cm]	**24 months** cc cavity [cm]	**36 months** cc cavity [cm]
P1	4	3,95	1,44	0,7	0,22	0,21
P2	3,7	4,05	1,47	0,73	0,25	0,18
P3	3,9	4,05	1,43	0,69	0,25	0,23
P4	3,7	4,1	1,35	0,7	0,25	0,18
P5	3,7	4,01	1,45	0,68	0,25	0,19
P6	4	4,05	1,44	0,73	0,22	0,21
P7	3,9	4,03	1,46	0,7	0,22	0,19
P8	4	4,02	1,41	0,68	0,25	0,17
P9	4,1	3,95	1,46	0,72	0,23	0,2
P10	3,9	3,97	1,42	0,71	0,26	0,15
P11	4,1	4,03	1,36	0,71	0,26	0,18
P12	3,8	3,98	1,46	0,69	0,24	0,19
P13	3,8	3,97	1,36	0,69	0,22	0,18
P14	3,9	4,05	1,45	0,71	0,25	0,15
P15	4	4,05	1,39	0,69	0,25	0,23
P16	3,9	3,95	1,42	0,68	0,26	0,15

P17	4,2	3,96	1,46	0,68	0,22	0,16
P18	4,2	4	1,35	0,71	0,24	0,23
P19	4,1	4,08	1,46	0,71	0,24	0,16
P20	3,8	4,03	1,41	0,7	0,24	0,23

Table S4. PSA trendline equations and R^{2}.

**Patients**	**Trendline equation**<br>y=PSA value<br>x=time	$\mathbf{R^2}$
P1	$y = 4,578e^{-0,027x}$	0,7055
P2	$y = 5,3203e^{-0,036x}$	0,888
P3	$y = 4,8583e^{-0,036x}$	0,7121
P4	$y = 5,3716e^{-0,043x}$	0,8184
P5	$y = 4,7833e^{-0,041x}$	0,7782
P6	$y = 5,3377e^{-0,036x}$	0,8191
P7	$y = 5,1919e^{-0,042x}$	0,928
P8	$y = 5,0906e^{-0,031x}$	0,8563
P9	$y = 5,2615e^{-0,031x}$	0,6683
P10	$y = 4,8527e^{-0,034x}$	0,709
P11	$y = 5,5395e^{-0,037x}$	0,8974
P12	$y = 5,5542e^{-0,033x}$	0,8216
P13	$y = 5,2138e^{-0,04x}$	0,8494
P14	$y = 6,0158e^{-0,048x}$	0,8918
P15	$y = 4,9516e^{-0,039x}$	0,8492
P16	$y = 4,9118e^{-0,031x}$	0,6966
P17	$y = 5,422e^{-0,034x}$	0,8169
P18	$y = 5,715e^{-0,036x}$	0,766
P19	$y = 5,3561e^{-0,035x}$	0,7794
P20	$y = 5,2017e^{-0,041x}$	0,8179

Table S5. Cavity cc volume trendline equations and R^{2}.

**Patients**	**Trendline equation**<br>y=cc cavity value<br>x=time	R^2
P1	$y = 3{,}0297e^{-0{,}088x}$	0,936
P2	$y = 3{,}0852e^{-0{,}09x}$	0,933
P3	$y = 2{,}9894e^{-0{,}085}$	0,9221
P4	$y = 3{,}0067e^{-0{,}089x}$	0,918
P5	$y = 3{,}0038e^{-0{,}088x}$	0,9291
P6	$y = 3{,}0778e^{-0{,}088x}$	0,9337
P7	$y = 3{,}0742e^{-0{,}09x}$	0,9378
P8	$y = 3{,}1088e^{-0{,}091x}$	0,9366
P9	$y = 3{,}105e^{-0{,}089x}$	0,9404
P10	$y = 3{,}1538e^{-0{,}094x}$	0,943
P11	$y = 3{,}1125e^{-0{,}09x}$	0,9304
P12	$y = 3{,}0339e^{-0{,}089x}$	0,9356
P13	$y = 2{,}9903e^{-0{,}091x}$	0,9311
P14	$y = 3{,}1884e^{-0{,}094x}$	0,943
P15	$y = 2{,}9929e^{-0{,}085x}$	0,9198
P16	$y = 3{,}124e^{-0{,}093x}$	0,9426
P17	$y = 3{,}1883e^{-0{,}095x}$	0,9489
P18	$y = 3{,}02e^{-0{,}085x}$	0,9203
P19	$y = 3{,}2257e^{-0{,}094x}$	0,9445
P20	$y = 2{,}9523e^{-0{,}085x}$	0,9202

Table S6. IPSS score.

Patients	0 month	36 months
P1	7	7
P2	6	7
P3	7	5
P4	8	3
P5	7	6
P6	9	8
P7	6	7
P8	5	2
P9	9	10
P10	10	11
P11	5	5
P12	7	7
P13	10	8
P14	7	6
P15	16	15
P16	4	3
P17	3	2
P18	3	3
P19	15	14
P20	3	5

Table S7. IEFF-5 score.

Patients	0 month	36 months
P1	7	7
P2	6	7
P3	7	5
P4	8	3

P5	7	6
P6	9	8
P7	6	7
P8	5	2
P9	9	10
P10	10	11
P11	5	5
P12	7	7
P13	10	8
P14	7	6
P15	16	15
P16	4	3
P17	3	2
P18	3	3
P19	15	14
P20	3	5
